# Anatomical study of the accessory axillary vein in cadavers: a contribution to the axillary surgical approach

**DOI:** 10.1590/1677-5449.003616

**Published:** 2016

**Authors:** Valtuir Barbosa Felix, José André Bernardino dos Santos, Katharina Jucá de Moraes Fernandes, Dhayanna Rolemberg Gama Cabral, Carlos Adriano Silva dos Santos, Célio Fernando de Sousa Rodrigues, Jacqueline Silva Brito Lima, Antônio José Casado Ramalho

**Affiliations:** 1 Universidade Federal de Alagoas – UFAL, Hospital Universitário Prof. Alberto Antunes – HUPAA, Departamento Anatomia Humana, Maceió, AL, Brazil.; 2 Centro Universitário CESMAC, Departamento Anatomia Humana, Maceió, AL, Brazil.; 3 Universidade Estadual de Ciências da Saúde de Alagoas – UNCISAL, Departamento Anatomia Humana, Maceió, AL, Brazil.; 4 Universidade Federal de Alagoas – UFAL, Departamento Anatomia Humana, Maceió, AL, Brazil.

**Keywords:** axillary vein, cardiac catheterization, anatomy, axilla, veia axilar, cateterismo cardíaco, anatomia, axila

## Abstract

**Background:**

The axillary vein is an important blood vessel that participates in drainage of the upper limb. Some individuals present a second axillary vein (accessory axillary vein), which is an important collateral drainage path.

**Objectives:**

The goal of this study was to determine the incidence of the accessory axillary vein and to describe this vessel’s topography.

**Methods:**

In this study, axillary dissections were carried out on twenty-four (24) human cadavers of both sexes that had been fixed with 10% formaldehyde. The upper limbs of the cadavers were still attached to the bodies and the axillary structures were preserved. Data collection was carried out and the axillary structures of the cadavers were compared.

**Results:**

The incidence of accessory axillary veins was 58.3%, with no significant preference for sex or for side of the body. The accessory axillary vein originated from the lateral brachial vein in 39.28% of cases, from the common brachial vein in 35.71% of cases, and from the deep brachial vein in 25% of cases.

**Conclusions:**

Its high incidence and clinical relevance make the accessory axillary vein important for provision of collateral circulation in the event of traumatic injury to the axillary vein.

## Resumo

**Contexto:**

A veia axilar é um importante vaso que participa da drenagem sanguínea do membro superior. Porém, em alguns indivíduos, é observada uma segunda veia axilar, denominada veia axilar acessória, que é uma notável via de drenagem colateral.

**Objetivos:**

O objetivo desta pesquisa foi observar a prevalência da veia axilar acessória, bem como descrever a topografia desse vaso.

**Métodos:**

Neste estudo foram realizadas dissecações das axilas em 24 cadáveres humanos fixados em formaldeído 10%, de ambos os sexos, que apresentavam os membros superiores articulados ao tronco e com as estruturas axilares preservadas. A coleta de dados foi realizada e as estruturas axilares dos cadáveres foram comparadas.

**Resultados:**

Foi encontrada uma prevalência de 58,3% de veias axilares acessórias, não havendo predileção significativa por gênero nem por lado acometido. Também foi observado que, em 39,28% dos casos, a veia acessória era originada a partir da veia braquial lateral, em 35,71% a partir da veia braquial comum, e em 25% a partir da veia braquial profunda.

**Conclusão:**

Devido à sua alta prevalência e relevância clínica, a veia axilar acessória também assume grande importância no tocante à circulação colateral diante de lesão traumática da veia axilar.

## INTRODUCTION

The axillary vein (AV) starts at the junction of the basilic vein with the lateral and medial brachial veins,^1^ or from the union of the common brachial vein, derived from the initial junction of the two brachial veins with the basilic vein.^2^^,^ 3 The AV can also originate from the direct and upward continuation of the basilic vein, which belongs to the superficial venous system.^4^

The accessory axillary vein (AAV) is a satellite axillary vein, and it can originate from the common brachial vein, the deep brachial vein, or even from the lateral brachial vein (in cases in which this vein does not join with the basilic vein or the medial brachial vein).^3^

Deep Venous Thrombosis of the upper limb mainly occurs in the axillary vein and subclavian vein,^5^ and in cases of obstruction, the AAV can act as an important collateral drainage path.^6^

The objective of this paper was to determine the incidence of the accessory axillary vein and to describe the topography of this vessel in the axillary region in adult cadavers.

## MATERIAL AND METHODS

This was an anatomical descriptive study of the axillary region of 24 human cadavers. A total of 48 axillae were dissected between 2013 and June 2014. The study was approved by the Human Research Ethics Committee at the Centro Universitário CESMAC, Maceió, AL, Brazil (CAAE: 17471813.5.0000.0039).

All cadavers were fixed in a 10% formaldehyde solution, the upper limbs were still attached to the bodies, and all structures pertaining to the axilla were well preserved.

The dissection was carried out with the cadaver in supine position, while the upper limb was abducted. After making a longitudinal incision in the arm and thorax median line, the skin was removed and muscles, vessels, and most superficial nerves were dissected to expose the axillary vessels.

The materials used were: scalpel handles numbers 3 and 8, scalpel blades (4 number 15 blades and 4 number 24 blades), straight and curved scissors, straight and curved hemostatic tweezers, rat-tooth and anatomical forceps, a photographic camera (Canon Powershot SX400IS digital camera), Netter’s atlas of human anatomy (6th edition), and Shearer’s Manual of Human Dissection (8th Edition).

Data collection was carried out and the axillary structures of the cadavers were compared.

## RESULTS

An AAV was present in 28 (58.3%) of the axillae studied, 16 (57.14%) in male and 12 (42.85%) in female cadavers.

Accessory axillary veins were only present in the left arms of 13 cadavers (43.42%), were only present in the right arms of 11 cadavers (39.28%), and were present bilaterally in 2 cadavers (14.28%) (Table 1).

**Table 1:** Incidence of accessory axillary vein in the cadavers studied.

**Location**	**Number of Cadavers**	**%**
Right axilla	13	46.42
Left axilla	11	39.28
Bilateral	2	14.28

With regard to formation of the AAV, it was observed that in 11 cases (39.28%) the AAV originated from the lateral brachial vein, in 10 (35.71%) cases from the common brachial vein, and in 7 (25%) cases from the deep brachial vein (Table 2). The AAV originated at the lower margin of the teres major muscle and ended in front of the subscapularis muscle at the level of the first rib.

**Table 2:** Veins from which the accessory axillary veins originated.

**Origin**	**Number of accessory axillary veins**	**%**
Lateral Brachial Vein	11	39.28
Common Brachial Vein	10	35.71
Deep Brachial Vein	7	25

**Figure 1:**
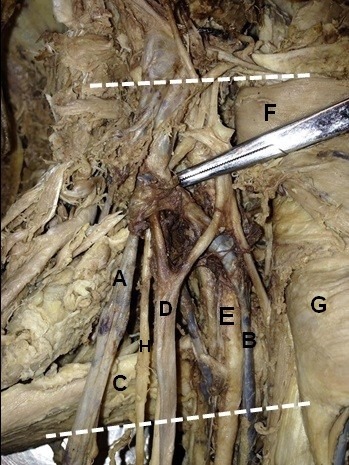
Right axilla, in (A) accessory axillary vein; (B) the axillary vein; (C) teres major muscle; (D) lateral fascicle; (E) axillary a.; (F) pectoralis minor m.; (G) pectoralis major m.; (H) musculocutaneous n.

The general topography and the trajectory of AAV was as follows: the AAV was lateral to the axillary artery in 42/48 regions (87.5%) and in 6/48 (12.5%) was posterolateral to the artery, as illustrated in Figures 1 [Fig gf02] [Fig gf03]-4. It ascended laterally to the brachial plexus in 44/48 (91.66%), while in the remaining 4/48 (8.33%) cases, the brachial plexus was absent because it had been removed during previous dissections. The vessel can also be situated between the musculocutaneous n. (anteriorly) and the axillary n. (posteriorly), as we observed in 21/48 (43.75%) cases. In 39/48 cases (81.25%), the AAV terminated in regions situated along an inferior portion of the AV, in 7/48 cases (14.58%) it terminated in the mid portion, and in 2/48 cases (4.16%) it ended at the subclavian vein.

**Figure 2 gf02:**
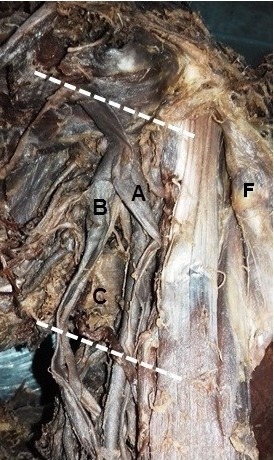
Left axilla, in (A) accessory axillary vein; (B) the axillary vein; (C) teres major muscle; (F) pectoralis minor m.

**Figure 3 gf03:**
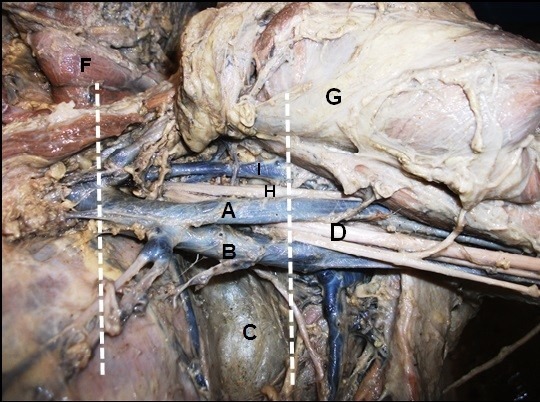
Left axilla, in (A) accessory axillary vein; (B) the axillary vein; (C) teres major muscle; (D) lateral fascicle; (F) pectoralis minor m.; (G) pectoralis major m.; (H) musculocutaneous n. and (I) cephalic v.

**Figure 4:**
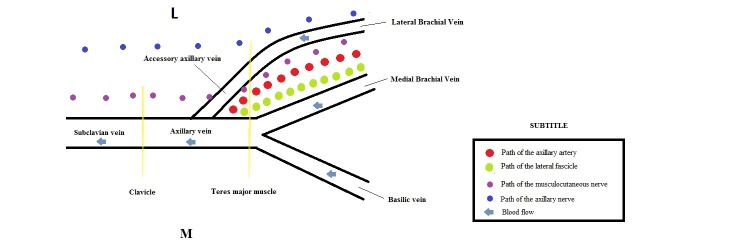
Schematic drawing of the general topography and trajectory of the accessory axillary vein (AAV).

With regard to cases with high numbers of tributary veins, 5 tributary veins of the AV were observed in 27 axillae (56.25%) and 3 tributaries of the AAV were observed in 13 axillae (46.42%).

The Langer axillary arch was not observed in any of the axillae studied.

With regard to the number of AV valves, 25 (52%) cases exhibited 7 valves, 14 (29.16%) cases had 9 valves, 5 (10.41%) cases had 4 valves, and 4 AVs (8.33%) had 6 valves. With regard to the number of AAV valves, 14 (50%) had 6, 8 (28.57%) had 7, 5 (17.85%) had 4, and 1 (3.57%) had 2 valves.

During dissection, it was observed that the axillary artery was located lateral to the AV in all of the cadavers. The lymph nodes related to upper limb drainage were situated just below the AV and the pectoralis minor muscle was located anterior to the axillary vein and artery. However, 39 (81.25%) of the axillae studied lacked at least one of the following structures: the intercostal brachial nerve (this is usually located medial of the axillary vessels), the long thoracic nerve (always next to the intercostal nerve), the medial cord of the brachial plexus, and the median, ulnar, and pectoral nerves (the latter are usually between the vein and the axillary artery). That is, only 18.75% of the axillae studied exhibited all of the structures listed above, which are usually observed in this region.

## DISCUSSION

The axillary vein has been widely discussed in the literature, with an increasing number of reports regarding its use in clinical and surgical procedures.

Gray (1988) and Gusmão (1992) report that the AV is formed by the junction of the basilic vein with the lateral and medial brachial veins, or by the union of the common brachial vein, which is formed by the initial junction of the two brachial veins with the basilic vein.^1^^,^ 2 However, according to Hollinshead & Rosse (1991) and later Gusmão in 2003, the AV is the direct and ascending continuation of the basilic, which belongs to the superficial venous system. The basilic vein perforates the brachial fascia and follows a path toward the axilla, where at this deeper point, it is called the axillary vein.^5^ Our results showed that the levels of the origin and termination of the axillary vein were similar to those reported by Gray (1988): it originates at the lower edge of the teres major muscles and terminates in front of the subscapularis tendon at the lateral margin of the first rib, at which point the axillary vein is provided with a pair of valves.

In this study, the incidence of accessory axillary veins was 58.3%. This result is similar to the 56.7% reported by Gusmão & Prates (1992).^6^ The AAV has often been described as a satellite vein of the AV or as a collector channel that drains the circumflex veins, which has led some researchers to name it as a small axillary vein, which at some point will flow into the true axillary vein and therefore should not be considered an anatomical variation, because it is not a rare finding.

In our study, we observed that 11 (39.28%) of the AAV found were formed by the lateral brachial vein, 10 (35.71%) by the common brachial vein, and 7 (25%) by the deep brachial vein. Gusmão & Prates (1992) found incidence rates of 55.9% of cases of AAV formed by the lateral brachial vein, 33.4% by the common brachial vein, and 11.8% by the deep brachial vein.^6^

Tributary veins were found in 27 (56.25%) axillae, 5 of which were tributary veins of the AV, and in 13 axillae (46.42%) there were 3 tributaries of the AAV. This suggests that these tributaries do not perform blood drainage from the same territories and are therefore not equivalent.

The valves of the axillary veins have been widely used to replace those of patients with chronic venous insufficiency.^5^^,^ 7 In our series, 25 AV (52%) had 7 valves and 14 AAV (50%) had 6 valves.

With regard to the likelihood of using the AAV for hemodialysis purposes, we believe that due to the difficult access and the depth of the vessel, this is highly unlikely. However, Lin et al. (2011), used ultrasound as a guide to the axillary vein and there are no reports in the literature with regard to use of ultrasound to locate the AAV.^8^

There are several reports in the literature about the possibility of deep vein thrombosis involving the axillary and subclavian veins.^3^^,^ 9 In such cases, the AAV would be an alternative pathway for venous drainage and restoration of blood flow to this region.^3^^,^ 10 ^,^ 11

The accessory axillary vein can also be of great importance for collateral circulation in the event of traumatic injury to the axillary vein.^9^ The larger the caliber of the AAV, the better it will function as a collateral alternative.^8^

## CONCLUSION

Our results allow us to conclude that the incidence of AAV was 58.3%, with no predilection for sex or body side, and the AAVs found often originated from the lateral brachial vein or the common brachial vein. There are few data in the literature regarding AAV incidence and its potential clinical and surgical applications. In view of its high incidence and clinical relevance, further studies are warranted.
